# Membrane–Ion Interactions

**DOI:** 10.1007/s00232-017-0010-y

**Published:** 2018-01-12

**Authors:** Ran Friedman

**Affiliations:** Department of Chemistry and Biomedical Sciences and Centre of Excellence “Biomaterials Chemistry”, Linnæus University, Kalmar, Sweden

**Keywords:** Molecular dynamics, Alkali ions, Specific ion effects, Quadrupole NMR, $^{32}\hbox {Na}$ NMR

## Abstract

Biomembranes assemble and operate at the interface with electrolyte solutions. Interactions between ions in solutions and the lipid affect the membrane structure, dynamics and electrostatic potential. In this article, I review some of the experimental and computational methods that are used to study membrane–ions interactions. Experimental methods that account for membrane–ion interactions directly and indirectly are presented first. Then, studies in which molecular dynamics simulations were used to gain an understanding of membrane–ion interactions are surveyed. Finally, the current view on membrane–ion interactions and their significance is briefly discussed.

## Introduction

### Interactions Between Ions and Macromolecules

Studies and theories that deal with the behaviour of macromolecules in electrolyte solutions date back to the nineteenth-century, if not before. One of the most interesting observations in this respect was made by Hofmeister (1888), who showed that different salts can solubilise or precipitate proteins and that the effect depends on the specific salt [for a review of Hofmeister’s works, see Kunz et al. (2004)]. At that time, Hofmeister was not familiar with the concept of ions, which was developed at the same period by Arrhenius. Nowadays, we tend to discuss specific ion interactions rather than the influence of salts. Such specific ion interactions may or may not follow Hofmeister’s series, and are not limited to proteins, or in fact to biomolecules (Friedman 2013). The polar head-groups of lipid membranes can directly interact with ions in solution, which in turn can influence the membrane’s properties. Ions in solution can affect lipid phase transitions (Träuble and Eibl 1974), modify the membrane potential (Hodgkin and Horowicz 1959), and alter the dynamics of the hydration layer (Song et al. 2014), to give a few examples.

### Lipids and Their Charges

Electrostatic solutions interact with membranes through opposing forces. On the one hand, purely electrostatic (charge–charge) and, to a much lesser extent, charge–dipole interactions favour the localisation of ions close to the membrane surface. This results in the formation of a diffuse electric double-layer (Bangham et al. 1965). On the other hand, entropy favours a more uniform distribution of the ions, and thus the resulting state depends much on the temperature (Markovich et al. 2018). Specific interactions will of course depend also on the lipid composition of the membranes. Many of the lipids that are commonly found in biological membranes are zwitterionic or charged (Table 1). The negatively charged group is usually a phosphate, with p*K*a $\le 3.0$. Phosphatidylserine (PS) has a more complex ionisation curve since the p*K*a of its carboxylate is 5.5, but is mostly −1 charged at physiological conditions.

**Table 1:** Common phospholipids and sphingolipids and their charges

Lipid	Charged groups			Overall charge
	Group 1	Group 2	Group 3	
Anionic lipids				
Phosphatidylserine (PS)	Phosphate (−)	Carboxylate (−)	Amine (+)	− 1
Phosphatidylinositol (PI)	Phosphate (−)			− 1
Cardiolipin	Phosphate (−)	Phosphate (−)		− 2
Gangliosides	*N*-acetylneuraminic acid (−)$^*$			− 1
Zwitterionic lipids				
Phosphatidylcholine (PC)	Phosphate (−)	Choline (+)		0
Phosphatidylethanolamine (PE)	Phosphate (−)	Ethanolamine (+)		0
Sphingomyelin	Phosphate (−)	Choline (+)		0

$^*$ Less commonly also *N*-glycolyl-neuraminic acid (−)

### Archaeal Lipids

**Fig. 1:**
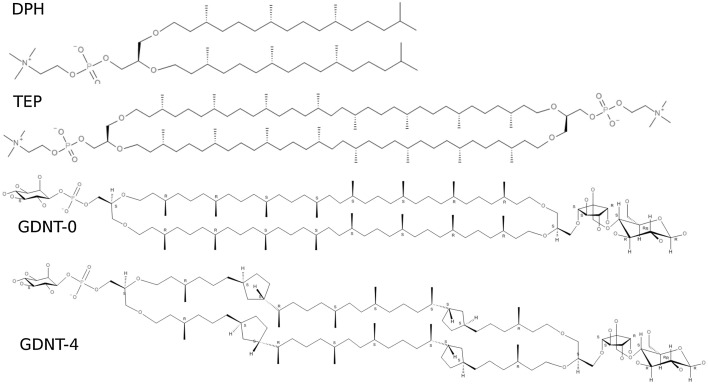
Examples of chemical structures of di- and tetra-ether archaeal lipids. DPhPC (DPH), ether-diphytanylphosphatidylcholine. TEP, di-O-biphytanyphosphatidylcholine. GDNT, glycerol dialkylnonitol tetraether, with zero (GDNT-0) or four (GDNT-4) cyclopentane rings. Reproduced from Pineda De Castro et al. (2016b) (Creative Commons Attribution License)

Archaeal membranes are chemically distinct from other biomembranes. The lipids that make those membranes do not have the typical di-esters that are almost uniformly common through other domains of life (bacteria and eukarya). Archaeal lipids linked to the head-group through *ether* rather than *ester* bonds, are branched rather than linear, and may have more complex structures (for some examples, see Fig. 1). Diether archaeal lipids form bilayers, similar to phosphodiesters. However, many archaeal lipids are tetraethers and form monolayers. Archaeal lipids are typically anionic or zwitterionic at one side and neutral at the other. The anionic group is typically a phosphate, and the cationic one is choline.

### Ions That Interact with Lipid Membranes

The ions that are most common in biologically relevant electrolytes are $$\hbox {Na}^+$$, $$\hbox {K}^+$$ and $$\hbox {Cl}^-$$. When studying specific ion effects, other alkali ions and halides are sometimes also considered. Some of these ions (e.g. $$\hbox {I}^-$$ and ^137^$$\hbox {Cs}^+$$) have medical significance, and others are used to examine the effect of size, polarisability or the ability to induce structure-ordering or structure-breaking of the water. SO_4_^2^$$^-$$, ClO_4_$$^-$$, and $$\hbox {SCN}^-$$ are also interesting in this respect. The first of these anions is a highly hydrated ion. Such ions have a large and favourable free energy of hydration. As a consequence, the water molecules close to them are highly structured (ordered), and the ions are referred to as water structure makers, or kosmotropes. ClO_4_$$^-$$ and $$\hbox {SCN}^-$$ are weakly hydrated ions. Such ions and the opposite effect to kosmotropes and are hence known as water structure breakers, or chaotropes. Finally, Ca^2+^ and Mg^2+^ are also of biological importance, and their interactions with the membranes can sometimes be of interest, in particular because multivalent cations can catalyse membrane fusion (Portis et al. 1979; Wilschut et al. 1980) or modify the membrane structure due to binding to multiple anionic sites on the membranes simultaneously.

## Experimental Methods Elucidate the Interactions Between Phospholipid Membranes and Ions

### Structural Studies of Phospholipid Membrane–Ion Interactions

**Fig. 2:**
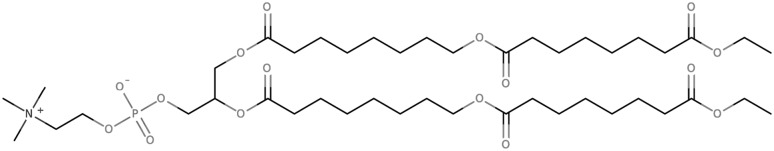
An ester-enriched phospholipid. Such ester-modified phospholipids were used to trap sodium ions by inside the membranes (through the use of ester-moieties), so that $^{23}\hbox {Na}$-NMR signals for internalised $\hbox {Na}^+$ could be recorded (Menger et al. 2006), which yielded a direct experimental demonstration of passive sodium transport

The binding of alkali cations, halogens, and even some larger anions (ClO_4_$^-$, $\hbox {SCN}^-$) or alkali-earth cations to lipid head-groups is transient, and it is therefore elusive to most experimental methods. One exception is NMR of quadrupolar nuclei. In a pioneering work, Lindblom used $^{23}\hbox {Na}$ NMR to show that $^{23}\hbox {Na}^+$ ions interact directly with membrane surfaces (Lindblom 1971). The same phenomenon was observed with all the alkali cations except for the very rare and radioactive francium, namely $^7\hbox {Li}^+$, $^{23}\hbox {Na}^+$, $^{39}\hbox {K}^+$, $^{85}\hbox {Rb}^+$, $^{87}\hbox {Rb}^+$ and $^{133}\hbox {Cs}^+$ (Lindblom and Lindman 1973). Competition experiments revealed that $\hbox {K}^+$ and $\hbox {Ca}^{2+}$ displaced $^{23}\hbox {Na}^+$ from binding sites on PS vesicles, whereas binding of the larger and more hydrophobic tetraethylammonium was disfavoured (Kurland et al. 1979). A demonstration of the usefulness of $^{23}\hbox {Na}^+$-NMR in the study of membrane–ion interactions was given about a decade ago, when the technique was used to reveal that sodium ion could be internalised in lipid membranes without the aid of ion-channels or carriers (Menger et al. 2006). The authors used ester-enriched modified lipids (Fig. 2) in order to detain the ions and observe an NMR signal from ions internalised inside the membrane.

A similar, though more indirect method to study how ions interact with lipid head-groups of biomembranes is $^{31}\hbox {P}$ or $^2\hbox {H}$ NMR. Deuterated lipids can be synthesised with deuterium in different positions, which has the advantage of showing how long membrane–ion interactions can perturb the quadrupole splitting. Moreover, such experiments yielded structural information on the orientation of the choline head-group (Seelig et al. 1987). $^2\hbox {H-NMR}$ measurements revealed that strongly hydrated anions (kosmotropes) had little effect on POPC membranes, which was also true for anions in the middle of the Hofmeister series ($\hbox {Cl}^-$ and $\hbox {Br}^-$, which are neither structure-making nor structure-breaking ions). Weakly hydrated anions bound to the lipids and perturbed the quadrupole of the choline. Interestingly, $\hbox {NO}_3^-$, which is between $\hbox {Cl}^-$ and $\hbox {Br}^-$ in the series interfered with the quadrupole moment but only for the $\beta$-carbon (the closest to the choline head-group). The weakly hydrated anions $\hbox {I}^-$, $\hbox {SCN}^-$ and $\hbox {ClO}_4^-$ had a strong effect on the two carbons that are closest to the choline (Rydall and Macdonald 1992).

Another method to estimate binding to lipid membranes is the use of solid supported membranes (SSM). In such experiments, a hybrid bilayer is made of surface (a gold electrode) bound alkanethiol and a lipid. The gold electrode can then be connected to a reference electrode, which enables the measurements of currents. SSM were used to infer on the binding of alkali, alkali earth and $\hbox {La}^{3+}$ cations as well as monovalent anions to biomembranes (Garcia-Celma et al. 2007). The results revealed that cation binding to PC was correlated to their hydration free energies, where kosmotropic ions (with large negative Δ$G_{\text {hyd}}$) displaced $\hbox {Na}^+$ ions more efficiently than chaotropic ones. The effect was roughly opposite for anions, i.e., chaotropic ions such as $\hbox {ClO}_4^-$ bound better to DPPC than kosmotropic ones such as $\hbox {F}^-$. In fact, charge displacement by $\hbox {Br}^-$, $\hbox {F}^-$ and $\hbox {SO}_4^{2-}$ was very close to zero. Apparent dissociation constants were estimated as 0.01, 0.34, 6, 27, and $>100\,\hbox {mM}$ for $\hbox {La}^{3+}$, $\hbox {Mg}^{2+}$, $\hbox {K}^{+}$, $\hbox {ClO}_4^-$, and $\hbox {Br}^-$, respectively. Infrared (IR) spectroscopy is also useful to shed light on the membrane structure and modifications to it e.g. as a function of temperature, stress or humidity. Interesting and complex ion-specific effects were measured in a study of the effect of metal-chloride solutions on the phase transition of POPC bilayers (Binder and Zschornig 2002). The bilayers underwent a gel to liquid-crystalline phase transition with increased relative humidity (RH). $\hbox {Na}^+$ and $\hbox {K}^+$ shifted the transition to lower RH values. $\hbox {Li}^+$ and most of the doubly charged ions that were studied (alkali earth metals and $\hbox {Cu}^{2+}$) shifted it to higher RH values, i.e. they stabilised the gel-phase. $\hbox {Zn}^{2+}$ inhibited transition at the experimental conditions. Interestingly, RH shifts were rather similar for $\hbox {Mg}^{2+}$, $\hbox {Sr}^{2+}$ and $\hbox {Ba}^{2+}$ and much more pronounced for $\hbox {Ca}^{2+}$ and $\hbox {Be}^{2+}$. This suggests that specific ion effects may involve not only binding of the cations to phosphates but also interactions with other groups.

### The Binding of Ions Affects the Potential, Dipole, Mechanical Stability and Dynamical Properties of Biomembranes

Studies of ion effects on the membrane are by no means limited to structural methods such as NMR. Many different biophysical properties of lipid membranes are perturbed by ion binding, and lessons learned from such studies help in devising theories that relate ion binding also to structural effects. The potential close to the membrane surface that includes also ions that bind to it ($\zeta$-potential) is very instrumental in understanding how ion binding affects the biologically relevant properties of membranes. $\zeta$-potentials of POPC vesicles were measured in alkali chloride solutions that varied in concentration from several tens to 500 mM (Klasczyk et al. 2010). The $\zeta$-potentials increased in concentrations up to 100 mM but not beyond. For reasons that are not entirely clear, POPC membranes have a slightly negative $\zeta$-potential (in this case, about $-\,8\,\hbox {mV}$). The potential became less negative in the presence of cations (it is positive only for $\hbox {Li}^+$ solutions, in concentrations above 50 mM). The order of the ion’s influence on the potential was $\hbox {Li}^+\,> \hbox {Na}^+\,>\,\hbox {K}^+ \approx \hbox { Rb}^+\,\approx \hbox { Cs}^+$. In a study by another group, where negatively charged DOPC and DOPG (4:1) vesicles were used, the order was $\hbox {Li}^+\,>\hbox { Na}^+\,>\hbox { K}^+\,\approx \hbox { Cs}^+>\hbox { Rb}^+$, and the membranes were not saturated in concentrations below 500 mM (Maity et al. 2016).

It has been suggested that ion binding to membranes may depend on the lipid type. For example, some types would bind better to kosmotropic anions than to chaotropic ones, whereas the opposite would be true for others (Leontidis et al. 2014). This, however, was only shown in Langmuir monolayers and not in bilayer membranes to the best of my knowledge.

One of the properties that can be perturbed by the binding of ions to the membrane surface is the dipole potential of the membranes. Lipid membranes carry a dipole potential, whose origin is believed to be the organisation of a few water layers at the membrane surface, and dipoles in the lipid (ester or ether groups, phosphates, cholines and the terminal methyls). If ions bind to the head-group, the dipole moment will change. Estimating the dipole moment from experiment is difficult and estimates vary as much as twofold between different methods (Wang 2012). It is nevertheless possible to infer on local variations in the potential by the use of amphipathic voltage-sensitive dyes. Such dyes shift their fluorescent excitation spectrum in response to changes in the electric field. A typical spectrum can be obtained with lipid vesicles in water and spectra in different salt solutions can be compared to the reference spectrum. Such experiments were carried out with zwitterionic DMPC vesicles (Clarke and Lupfert 1999). Interestingly, NaCl had no influence on the fluorescence of the voltage-sensitive dyes. Weakly hydrated anions such as $\hbox {ClO}_4^-$ or $\hbox {I}^-$ and strongly hydrated cations ($\hbox {Li}^+$ and multi-charged cations) had the largest effect on the fluorescence. A possible interpretation of these results is that cation effects are due to binding to the phosphate groups, whereas anion effects were due to partitioning of the anions between the membrane and aqueous phases.

A direct estimation on the strength of the ion binding to membranes can be provided by isothermal titration calorimetry. This is a more direct measurement than relying on potentials. Indeed, whereas $\zeta$-potential measurements of POPC membranes in alkali chloride solutions could not discriminate between $\hbox {K}^+$, $\hbox {Rb}^+$ and $\hbox {Cs}^+$, isothermal titration calorimetry (ITC) measurements showed clearly that the binding of the alkali ions depended on their size (the smaller the cation the higher was its affinity to the vesicles) (Klasczyk et al. 2010). Interestingly, the binding reaction was endothermic. Apparently, it is the entropic loss of the hydration shell water that drives binding, while the same process makes the enthalpy of binding positive (since the interactions of water molecules with the ions are favoured).

As ions that bind to the membrane modify the lipid structure, they also affect the mechanical properties of membranes. These properties can be estimated by experimental methods such as atomic force microscopy (AFM), electron spin resonance (ESR), deuterium NMR, calorimetry, and others. In particular, AFM was used to study how alkali and alkali-earth cations affect the stability of phospholipid membranes (Redondo-Morata et al. 2012). In general, the mechanical stability of lipid membranes increases with the ionic strength, because ions bind to the water-exposed head-groups thereby reducing electrostatic repulsion and consequently also increasing the vdW attraction between the hydrophobic tails. Interestingly, the force required to rupture the membrane by ATM increased with the ion size when the effects of $\hbox {Li}^+$, $\hbox {Na}^+$ and $\hbox {K}^+$ were studied. $\hbox {Cs}^+$ and the alkali-earth ions did not have a strong effect on the force when membranes were in their liquid-crystalline phase. In the gel phase, $\hbox {Cs}^+$ had no effect whereas the force increased with the ion size from $\hbox {Mg}^{2+}$ to $\hbox {Ca}^{2+}$ but was much smaller for $\hbox {Sr}^{2+}$. These measurements are thus interesting and difficult to interpret, as different effects come into play. Apparently, $\hbox {Li}^+$, $\hbox {Na}^+$ and $\hbox {K}^+$ bound to the PC membranes whereas $\hbox {Cs}^+$ did not bind appreciably to the membranes. Binding of the cations lead to binding of counter-anions ($\hbox {Cl}^-$ in this experiment). This effect was less pronounced for $\hbox {K}^+$ which may contribute to the higher resistance of the membranes in KCl solutions. The same argumentation can be used for the binding of alkali earth cations to the membrane in the gel-phase. In the liquid-crystalline phase, $\hbox {Cl}^-$ ions may follow both $\hbox {Mg}^{2+}$ and $\hbox {Ca}^{2+}$, but the molecular picture is far from clear.

## Simulations of Phospholipid Membranes and Ions Yield an Atomistic Understanding of Their Interactions

### Computer Simulations of Lipid Membranes

Biophysical methods are indispensable in studies of biomembranes and other biomolecules. Spectroscopy and calorimetry yielded data that are crucial to our understanding of membrane–ion interactions from a structural, energetic and even dynamical point of views. By and large, however, experimental methods are still not able to provide a detailed atomistic picture of membrane ion interaction and dynamics. Computer simulations can complement these methods by shedding light on timescales and system studies that are not available to the experiment (Friedman et al. 2013).

Attempts to model lipid membrane dynamics by use of computer simulations were made already in the late 1970s (Cotterill 1976). Reports on membrane simulations were few and far between until the 1990s. With the advancement of hardware and software, the field has revived. Today, web-based tools such as CHARMM-GUI (Jo et al. 2009) and computer programs such as VMD (Humphrey et al. 1996) (through the membrane plugin) simplify the construction of membrane systems, and even the addition of ions. Moreover, parallel computers, graphical processing units, and excellent computer simulation programs make membrane simulations routine today, although limitations on the accuracy remain. The simulations rely on approximated representations of the atom–atom interactions (force-field-based energy functions) and most implementations neglect multipoles, polarisation, many-body interactions and quantum effects.

### A Simulation Approach to Membrane–Ion Interactions

**Fig. 3:**
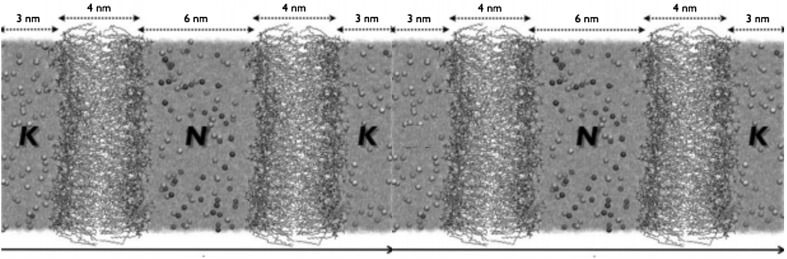
Set-up for a double-bilayer simulation of system with two different salt solutions. The bilayers (licorice representation) separate the solvent and ions ($\hbox {Na}^+$, N or $\hbox {K}^+$, K) in a periodic system (two copies are shown, on the *Z*-axis. Reproduced from Ref. Lee et al. (2008), with permission from Elsevier

The development of force-field parameters to accurately model common lipid membranes and alkali-chloride ions in solutions generated interest in modelling how salt ions affect the membrane structure and dynamics. Early studies followed biomembranes in water and in salt solutions, to reveal the effects of salts on the membrane’s electrostatic potential, structure and dynamics (Pandit et al. 2003; Böckmann et al. 2003). Once it was possible to model membrane–ion interactions fairly accurately, specific-ion interactions came into play. It is well known that the distribution of $\hbox {Na}^+$ and $\hbox {K}^+$ ions is asymmetric with respect to the cell membrane. The concentration of $\hbox {K}^+$ is higher in the cytoplasm of eukaryotic cells than in the extracellular milieu, while the opposite is true for $\hbox {Na}^+$. The membrane potential is negative when measured in the interior of cells with respect to the exterior, which was suggested to be due to preferential binding of $\hbox {Na}^+$ to the phosphate moieties of the membranes generating a less negative surface. This was studied by computer simulations with two DPPC bilayers that separated NaCl and KCl solutions in a periodic cell (Fig. 3). Upon equilibration, $\hbox {Na}^+$ ions indeed bound to the membrane surface much more than $\hbox {K}^+$, which generated a potential of $-\,70\,\hbox {mV}$, a result that is within the experimentally measured range (Lee et al. 2008). In another study, a double layer was used to mimic the cell membrane, this time with zwitterionic lipids outside and anionic lipids inside. $\hbox {Na}^+$ counterions were located in the compartment that mimicked the inner part of the cell. The cations bound to the membrane, but not enough to neutralise the charge, which resulted in an intrinsic membrane potential (Gurtovenko and Vattulainen 2008). One may expect that the next step would be to simulate an asymmetric double-bilayer system (with excess negative charge in the interior) and physiological concentration of ions. Such complex simulations were indeed performed, and revealed that the lipids bind primarily to $\hbox {Na}^+$ in the interior of the double-bilayer system (Vácha et al. 2009a).

### Specific Ion Effects

Specific ion effects are of course interesting beyond the context of the physiological concentrations of $\hbox {Na}^+$ and $\hbox {K}^+$. MD simulations can be used to calculate a variety of interesting properties that illustrate the effects of ions on the membrane structure. For example, mean residence times were calculated for alkali chloride solutions and were in the order $\hbox {Na}^+>\hbox { K}^+>$ $\hbox {Cs}^+$ (Vácha et al. 2009b). Interestingly, the residence times depend on the counter ions as well, e.g. they are shorter for $\hbox {Na}^+$ if the counter ion is $\hbox {Cl}^-$ than if it is $\hbox {Br}^-$ or $\hbox {I}^-$, because $\hbox {Cl}^-$ ions bind better to the already $\hbox {Na}^+$-bound membrane. In a later study, it was shown that $\hbox {I}^-$ has a similar if not higher affinity to DOPC membranes than $\hbox {Cl}^-$ (Vácha et al. 2010). The difference with respect to earlier finding was accounted to the counter ion ($\hbox {K}^+$ vs. $\hbox {Na}^+$) and the use of a polarisable force field, as polarisability contributed to the higher affinity of $\hbox {I}^-$ to the membrane. Indeed, ITC measurements have later shown that another PC membrane (DMPC) binds NaI better than NaCl (Wang et al. 2011).

By and large, the binding of ions to the membrane (studied by simulations mostly with alkali cations) is in line with the Hofmeister series. Strongly hydrated ions ($\hbox {Li}^+$ and multivalent ions) bind to the membranes more than weakly hydrated ones such as $\hbox {Cs}^+$. However, some phenomena do not follow Hofmeister series, and one example is membrane fluidity in salt solutions. Simulations of POPC membranes revealed that physiological chloride salt solutions reduced the fluidity of lipid membranes, as indicated by their lateral diffusion coefficient, in the order $\hbox {Na}^+>\,\hbox {K}^+>\,\hbox {Ca}^{2+}$ (Kagawa et al. 2013). Apparently, the cations introduce lipid dehydration when present at the membrane–water interface. Thus, it is not direct ion binding to the membrane that influences the membrane’s lateral diffusion coefficient but rather the cations’ presence close to the membrane–water interface. Free energy profiles calculated for the binding of ions to the membrane corroborate these findings as they also show that $\hbox {Ca}^{2+}$ ions bind strongly to the membrane while $\hbox {Na}^+$ and $\hbox {K}^+$ are likely to be hydrated at the interface (Yang et al. 2015).

Another interesting phenomenon can be described as specific lipid effects. Clearly, positive ions bind preferentially to negatively charged membranes, and anions avoid them. Cardiolipins are doubly anionic and hence have an increased affinity to cations. Interestingly, it has been shown in an MD study that membranes containing 9.2% cardiolipin and 91.8% POPC have a greater affinity to $\hbox {Na}^+$ cations than pure POPC or pure cardiolipin membranes (Dahlberg and Maliniak 2008). This may be due to such membranes being less stiff, as shown by their lower compressibility modulus.

### Interactions of Archaeal Membranes and Ions

Much less is known on archaeal membranes than on phosphodiester membranes. There are different types of archaeal lipids, and force field parameters for most are not available. Moreover, the tools that can be used to build a membrane structure prior to equilibration are not tailored to di- or tetra-ether membranes. Nevertheless, computational studies of archaeal membranes have been reported, and few studies focused on their interactions with ions.

In a study of DPhPC (di-ether) membranes, it was shown that $\hbox {Na}^+$ ions penetrated into the ether-rich region and formed salt bridges between two and even three lipids. This reduced the area per lipid (APL), but only for very concentrated solutions (4 M NaCl) (Shinoda et al. 2007). In the case of tetra-ether lipids, it was shown that large counterions modified the lipid structure when they bound to membrane. Already when only counterions were present (and no additional anions), the APL depended on the size of the cations (Pineda De Castro et al. 2016b). This was later corroborated by studies in NaCl and KCl solutions of different concentrations (Pineda De Castro et al. 2016a). Interestingly, the interaction free energy between the cations and the lipids was found to be in the order of $-\,1\,\hbox {kcal}/\hbox {mol}$ in low ion concentrations, and the long residence times suggested that the membrane act as an antenna, holding the ions close to the surface. This suggested that ions are ready to be transferred to the cells if necessary, enabling the survival of archaea in solutions that are nearly free of salts (Buetti-Dinh et al. 2016). It remains to be seen if the same antenna-like mechanism operates also for phosphodiester membranes.

### Important Considerations for Simulations and Analysis of Ions with Lipids

Simulation studies are only as accurate as the underlying force field and simulation method. In the case of membranes and ions, an accurate treatment of the lipids, ions and water as well as their interactions is required. In particular, anyone who runs such simulations should make an effort to verify that the ion-phosphate interactions are indeed representative. $\hbox {Cl}^-$, $\hbox {Na}^+$ and $\hbox {K}^+$ ions are generally easier to model, and there are several ion-water combinations that work. Smaller ions such as $\hbox {F}^-$ and $\hbox {Li}^+$ (Becconi et al. 2017) induce polarisation of the water, and their binding to lipids may be over- or under estimated. Larger monovalent ions (especially anions) can also be tricky to simulate due to their softness—they can in fact be somewhat more hydrophobic and polarisable. Multivalent ions are often more complicated and necessitate the use of polarisable force-fields or other non-conventional schemes. It has been shown that even $\hbox {Ca}^{2+}$ ions are difficult to represent correctly (Project et al. 2008), whereas transition metal and group XII ions such as $\hbox {Zn}^{2+}$ and $\hbox {Cd}^{2+}$ are challenging to model even with quantum chemistry (Ahlstrand et al. 2013, 2017).

There are limitations associated with the membrane models as well. One example is diffusion coefficients, which may deviate from experimental values due to the size and shape of the simulation box and the force field used (Camley et al. 2015; Venable et al. 2017) [of note, the diffusion coefficients of the ions may depend on the water model used in the simulations (Friedman et al. 2005)]. In simulations of membranes in an NaCl solution, overbinding of $\hbox {Na}^+$ was observed, which has been corrected in the CHARMM force field (Venable et al. 2013). Many other artefacts have been observed throughout the years [for a fairly recent review, see Wong-Ekkabut and Karttunen (2016)]. Overall, however, membrane simulations are most often accurate enough to be useful, as long as they are run carefully and with the up-to-date force fields and standards.

Another caveat of studies that follow on the effects of ion binding on the membrane structure is that the counter ion can also have some effects, as shown in simulations of proteins (Friedman 2011). If, for example, one wishes to compare the effects of different ions, the same counterion should be used in all cases otherwise cation–anion and counterion–membrane interactions can interfere with the analysis. This is especially important when comparing results from different measurements published in the literature. Similarly, due to screening and dynamics of the ions, ionic concentrations and temperatures are also important. Obviously, the temperature governs the membrane phase as well.

## Conclusions

Studies of membrane–ion interactions improved our understanding of membrane structure and physiology. Weakly hydrated anions bind to zwitterionic membranes better than strongly hydrated ones, which sometimes do not bind at all. Cations bind to anionic and zwitterionic membranes, but their binding patterns are more complex and not completely resolved. Simulations and some measurements (e.g. SSM potentials, spectroscopy and ITC) showed stronger binding for strongly hydrated cations, but secondary effects such as the cations’ influence on the membrane phase, fluidity and stiffness do not always follow the Hofmeister series, as demonstrated by measurements as well as simulations. Finally, correctly accounting for membrane–ion interactions, in simulations and experiments, is important not only for studies that aim at understanding the interplay between the ions and the membrane. Salts affect the membrane bilayer structure and thereby the interaction of the membrane with other molecules, including proteins and peptides.
